# Are the folk utilitarian about animals?

**DOI:** 10.1007/s11098-022-01833-2

**Published:** 2022-08-03

**Authors:** Guy Kahane, Lucius Caviola

**Affiliations:** 1 Uehiro Centre for Practical Ethics, University of Oxford, Oxford, England; 2 Department of Psychology, Harvard University, Cambridge, U.S.

**Keywords:** Animal Ethics, Moral Status, Hybrid view, Moral psychology, Robert Nozick, Deontology.

## Abstract

Robert Nozick famously raised the possibility that there is a sense in which both deontology and utilitarianism are true: deontology applies to humans while utilitarianism applies to animals. In recent years, there has been increasing interest in such a hybrid views of ethics. Discussions of this Nozickian Hybrid View, and similar approaches to animal ethics, often assume that such an approach reflects the commonsense view, and best captures common moral intuitions. However, recent psychological work challenges this empirical assumption. We review evidence suggesting that the folk is deontological all the way down—it is just that the moral side constraints that protect animals from harm are much weaker than those that protect humans. In fact, it appears that people even attribute some deontological protections, albeit extremely weak ones, to inanimate objects. We call this view Multi-level Weighted Deontology. While such empirical findings cannot show that the Nozickian Hybrid View is false, or that it is unjustified, they do remove its core intuitive support. That support belongs to Multi-level Weighted Deontology, a view that is also in line with the view that Nozick himself seemed to favour. To complicate things, however, we also review evidence that our intuitions about the moral status of humans are, at least in significant part, shaped by factors relating to mere species membership that seem morally irrelevant. We end by considering the potential debunking upshot of such findings about the sources of common moral intuitions about the moral status of animals.

## Introduction

The vast majority of people regard humans as vastly more important than (non-human) animals. This is manifested in a wide range of routine acts and practices, such as meat consumption, animal experimentation and hunting. And people believe that these acts and practices are morally permissible—for many, they are so obviously correct that they don’t even need justification. We will call this view *Moral Anthropocentrism*: the view that it is morally permissible (or even obligatory) to prioritise humans over other animals when the interests of humans and animals conflict. In one extreme form of Moral Anthropocentrism, humans matter much more than animals simply because animal lack any moral status, and we can permissibly do anything whatsoever to animals if we so desire. While something like this view was probably widespread in the past, most people today would reject it, holding that animals do matter morally but far less so than humans. What, however, does it mean for animals to matter morally, yet matter less?

One answer to this question was suggested (though not endorsed) by Robert Nozick a while ago. On this view, whereas humans are protected by rights, such as the right not to be killed, animals have only interests (cf. Feinberg, 1974), interests which we should aim to promote, or even maximise, even if that requires us to sacrifice some to promote the greater good. Nozick captured this idea with the memorable slogan, ‘Kantianism for people, utilitarianism for animals’ (Nozick, 1974). Some prominent moral philosophers have endorsed something like this idea. For example, Judith Jarvis Thomson remarked in passing that.

“animals do not have claims to not be killed and, in particular, … it is not an infringement of any claim of a chicken’s to kill it for dinner. (Would it be permissible to kill one chicken to save five chicken? I think it would.)” (Thomson, 1990, 292).

And after remarking that “[i]t is uncontroversial that the killing of an animal is normally less seriously wrong than the killing of a person,” (McMahan, 2002, 190) Jeff McMahan develops a two-tiered account of the wrongness of killing that divides morality into the morality of respect and the morality of interests. Since people are autonomous and rational, they are entitled to respect, and thus to moral protections against being killed or used, even when this would lead to an optimific result, protections that non-persons (including all or most animals) do not enjoy.

In recent year, there has been increasing interest in such Nozickian Hybrid Views that see deontology as applying in the domain of humans and consequentialism as applying in the domain of animals (or, in McMahan’s case, those of persons and non-persons). Even those, such as Shelly Kagan, who go on to reject such theories, hold that they are “worth taking very seriously” because they are highly intuitive (Kagan, 2019). For example, to illustrate the ‘intuitive appeal’ of the Nozickian View, he asks us to consider.

“[a] case where we can save five people, but only by killing a sixth. As we know, most find this unacceptable… But I imagine that most of us would have a rather different reaction to this sort of case if it involved animals rather than people. Suppose, for example, that we can save five rabbits, but only by killing a sixth rabbit. Here, I suspect, a deontological reaction won’t seem appropriate at all. On the contrary, most will comfortably conclude that while it is unfortunate that we cannot save the five rabbits without sacrificing the sixth, still, on balance, killing the one rabbit is indeed the right thing to do, since this is the only way to save a larger number of rabbits overall…. In short, when it comes to thinking about animals most of us are quite comfortable thinking in consequentialist terms.” (Kagan, 2019, 193).

Kagan hesitates to describe such a Nozickian theory—which he calls ‘restricted deontology’—as the ‘commonsense view of the matter’. But this is largely because he doubts that ordinary folk have any sophisticated view on this issue. But he thinks that the Nozickian View clearly captures the moral view implicit in commonsense: he writes that it “represents a rational reconstruction of the moral outlook that may lie behind a great deal of common moral thinking.” (Kagan, 2019, 194).

Killoren & Streiffer (2019) similarly hold that the Nozickian View deserves serious exploration because it captures key aspects of ordinary practice—for example, most people are comfortable with zoos or animal experimentation, so long as these have a benefit to humans and animals’ welfare is taken into account, whereas such practices would seem outrageous if they involved humans. And they point out that many organisations and institutions explicitly appeal to consequentialist principles when assessing when harm to animals is permissible. For example, they point out that utilitarian cost-benefit analysis serves as ‘‘the cornerstone’’ of animal research regulations in the UK (Nuffield, 2005: 27, 52).

We will not be directly concerned here with whether the Nozickian View is the best way to develop Moral Anthropocentrism, or with whether Moral Anthropocentrism is itself defensible. Instead of directly addressing these normative questions, we will consider an important descriptive question: are Kagan, Killoren and Streiffer correct in thinking that the Nozickian View captures the intuitive, commonsense view of the matter—and that the Nozickian View therefore also likely underlies many current practices involving the use of animals?

In the first part of this paper, we will review new evidence from experimental work we have conducted in order to address this question, and to generally shed light on the psychology of Moral Anthropocentrism (largely drawn from Caviola, et a., 2021).^1^ While recent work in moral psychology is sometimes claimed to directly undermine, or support, major ethical theories such as deontology or utilitarianism (see e.g., Greene 2008), we doubt that empirical findings can conclusively settle grand questions in ethical theory in this way (Kahane, 2016; Everett & Kahane, 2020). We do think, however, that the research we will describe is of considerable philosophical interest. One way in which such research has obvious normative relevance is by directly testing the current assumption that the Nozickian View reflects intuitive commonsense.^2^ We will present empirical evidence suggesting that this assumption is mistaken: most people are deontological all the way down.

Despite regarding it intuitive, Kagan rejects the Nozickian View because he thinks that the sharp moral distinction it requires between humans (or even just persons) and other animals cannot be sustained.^3^ He therefore argues that those who are attracted to deontology ought to extend it to cover animals as well—it’s just that the deontological protections that animals enjoy are weaker than those protecting humans. We will present empirical evidence suggesting that something like this view *just is* the intuitive, commonsense view. We will then consider whether these findings undermine the Nozickian View, and whether they instead support the kind of hierarchical deontological view that Kagan describes, which we will call *Multi-level Weighted Deontology*.

Another way in which empirical findings about moral psychology can have potential normative significance is by uncovering the factors and processes that underlie some intuitive moral conviction or distinction (Kahane, 2013). While our focus will be on how ordinary folk conceive of the moral difference between humans and non-human animals, we will also consider questions about the factors that psychologically underlie this common moral distinction—is Moral Anthropocentrism based in the perception that humans have, say, greater cognitive capacities, or does it reflect, as Peter Singer argues, a mere preference for members of our own species (aka ‘speciesism’)?^4^ Also relevant here is the developmental basis of Moral Anthropocentrism—is it hardwired or is it acquired and, if so, how? We will review novel empirical evidence suggesting that full-blown Moral Anthropocentrism probably emerges only in early adulthood (Wilks et al., 2021) and that it is partly—though not entirely—driven by concern with bare species membership and other factors that seem morally irrelevant (Caviola et al., 2021), and we will again consider what normative difference, if any, these further empirical findings might make.

## Testing the Nozickian Hybrid View as a descriptive hypothesis

### Possible views about the permissibility of harming humans and animals

As we saw, the Nozickian approach is often presented as highly intuitive and as capturing what underlies the commonsense view. This is an empirical claim: are folk moral judgments really best captured by the slogan ‘deontology for people, utilitarianism for animals’? This slogan is clearly at least half right. Its first part—the part concerning humans—has been investigated extensively over the past two decades. Numerous studies, largely relying on the trolley dilemma paradigm (henceforth, ‘sacrificial dilemmas’) have repeatedly demonstrated that most people are deontologists in the sense that they often regard it as wrong to harm or kill some innocent people even when that would lead to the best outcome—e.g., saving the lives of many others (e.g., Greene 2013). Although this may not be how they conceptualise this, most (but not all) people accept strong deontological constraints forbidding such harm. So ‘deontology for people’ is descriptively correct—though, importantly, ‘*Kantianism* for people’ probably is not, since many (but not all) people regard these constraints as defeasible if the consequences of refraining from such harm are severe enough (Caviola et al., 2021). And it is worth nothing that a sizeable minority *is* happy to apply consequentialism to people, at least in the context of questions about harm.

The interesting question, then, is whether people also accept ‘utilitarianism for animals’.^5^ When they provide evidence that something like this is the commonsense view, Killoren and Streiffer point out that most people regard it permissible to treat animals in ways we would regard as deeply disrespectful if humans were involved, even if the humans were otherwise happy and well provided—think, for example, of zoos, or owning a pet. As mentioned earlier, they also point out that the regulation of animal research and meat production focus on utilitarian considerations relating to the welfare of animals and benefit to humans while such research on humans (let alone cannibalism) is strictly prohibited on deontological grounds. However, Killoren and Streiffer concede that many people would still see certain ways of treating animals as degrading even if they don’t harm these animals. And the permissibility of harming animals to benefit humans, while compatible with consequentialist considerations, can also be accommodated by hierarchical deontological views on which animals enjoy *some* deontological protections but these can be overridden when the interests of humans—beings with superior moral status—are at stake.

We therefore follow Kagan and Thomson in seeing the intra-species context as a crucial test case: most people reject the idea of sacrificing one human to save five, but would they see it permissible to sacrifice one animal to save five others? Thomson answers in the affirmative, and Kagan assumes that so would most others. Whether this is so is what we investigated in a series of studies that we will summarise below.

Before we do so, however, it will be useful to sketch a general framework for interpreting the findings we will report. In order to delineate the space of possible views about the permissibility of harm to humans vs. to animals, we will briefly outline six ethical theories that offer distinctive answers to this question (Table 1). Three of these—utilitarianism, classical Kantianism and what we call ‘Cross-Species Deontology’—are clearly not descriptively adequate and included only for the sake of completeness. The three others, however, are at least prima facie compatible with familiar practices and judgments.

The first view is *Utilitarianism*. As we will understand it here, on this view equal harms matter equally, regardless of who suffers them—whether humans or animals (Bentham, 1780). This view is anti-speciesist, meaning that species membership itself should not influence the moral status of an individual (Regan & Singer, 1989). If people were strict utilitarians, they would consider it permissible (or even required) to sacrifice both humans and animals to promote the greater good (of both humans and animals). It is unlikely, however, that this view captures the intuitions of most people since, as described above, most people accept deontological constraints against harming humans for the greater good (e.g., Greene 2013), and people generally tend to value animals less than humans (Caviola et al., 2019). However, since a minority *is* willing to engage in consequentialist cost-benefit analysis in the context of harm to humans, it’s likely that this minority will continue to do so in the animal context.

A second view is *Kant’s* own view. On this account, only humans matter morally and therefore deserve deontological protection, whereas animals are just seen as objects that can be used to our own ends, though gratuitous harm to animals is forbidden because it could lead to harm to humans (Kant, 1797/2017, 6:433). This, however, is also implausible as an account of most people’s view since people do believe that animals matter morally at least to some extent (Caviola et al., 2019).

The third view, which we call *Cross Species Deontology*, says that the same deontological principles apply in the same way to all species: neither humans nor animals should be sacrificed for the greater good of either (for a broadly similar view, see Regan 1987).^6^ While some animal rights activists endorse similar moral positions (e.g., Francione 1995), this view is again unlikely to capture the commonsense view given that most people hold that it is permissible to harm animals to benefit humans, e.g., via medical testing (Caviola et al., 2019). In fact, a recent large-scale study employing sacrificial dilemmas involving autonomous vehicles confirmed, unsurprisingly, that willingness to sacrifice animals to save human lives is overwhelmingly endorsed by most people around the world (Awad et al., 2018)

There are, however, at least three ways to capture this intuitive moral difference between humans and animals while still ascribing some moral significance to animals. One is the *Nozickian View* we already discussed above on which deontology applies only to humans, while consequentialism applies to animals. On this view there is no intrinsic moral reason not to sacrifice one animal to save five others of the same species. The two last views extend deontology all the way down. According to what we call *Multi-level Uniform Deontology*, there is a hierarchy of moral status and individuals that are lower in the hierarchy (e.g., pigs) can be sacrificed for the sake of those higher up (e.g., humans). But *within* each level of moral status, the deontological constraints offer the same protections (i.e., it’s generally wrong to sacrifice a pig to save five pigs), and these protections apply to the same degree. Finally, according to what we call *Multi-level Weighted Deontology*, deontological protections are not absolute, and get weaker the lower the level of moral status. As we go down the hierarchy, the less stringent the deontological constraints. This is similar to the hierarchical view that Kagan recommends to deontologists^7^ and is also perhaps what Nozick himself gestures towards when he expresses his dissatisfaction with what we here call the Nozickian Hybrid View (which, to repeat, was not Nozick’s own view!).

As an empirical hypothesis about ordinary people’s moral judgments, Multi-level Weighted Deontology (henceforth, MLWD) predicts that people will consider harming animals to save many animals neither completely permissible nor completely wrong, but instead somewhere in between. Further, the lower the moral status of the animal in question, the more permissible they would consider harming it to save many animals with the same moral status (i.e., it is more permissible to sacrifice one cow to save five others than to sacrifice one human to save five others). In cases where the moral status of a being is perceived as very low (as for example with very simple animals), the implications of the MLWD will resemble those of the Nozickian View because the deontological constraints will be low or non-existent.

**Table 1:** Moral views about the permissibility of harming humans and animals

Harm: To save:	Humans Humans	Animals Animals	Animals Humans	Humans > Animals
Utilitarianism	✓	✓	✓	✕
Kant’s view	✕	✓	✓	✓*
Cross-species Deontology	✕	✕	✕	✕
Nozickian Hybrid View	✕	✓	✓	✓
Multi-level Uniform Deontology	✕	✕	✓	✓
Multi-level Weighted Deontology	weighted	weighted	✓	✓

*Note*. Humans > Animals stands for humans have higher moral status than animals (even if they are equally sentient)

* While Kant thought that the moral status of animals is not fundamentally different from that of objects, he did think that harming animals gratuitously is wrong on instrumental grounds, because it can make us more willing to harm humans

### Evidence for multi-level weighted deontology

In a series of experiments, we empirically investigated which of the above moral views best captures the intuitions of non-philosophers. As Kagan rightly points out, most people don’t have sophisticated theoretical views on these questions so asking them directly to endorse or reject explicit views about moral status is unlikely to shed much light on the structure that may be implicit in people’s moral judgments about more concrete cases involving humans and animals. There is in fact considerable evidence that the more general principles that lay folk are willing to endorse often have only limited relation to their more concrete judgments Cushman et al., 2006; Hauser et al., 2007; Lombrozo, 2009). We therefore primarily investigated people’s moral judgments about a range of specific dilemmas, in line with most current research in moral psychology.^8^ Our question, then, was which of these general theoretical frameworks best captures that pattern of judgments that ordinary people make about a range of relevant case—but in no way assuming that something like such a theoretical framework consciously guides these judgments. Although the trolley-inspired sacrificial dilemma paradigm has its limitation, it is by far the most widely used paradigm for studying people’s judgments about harm in the human context. For that reason, and to allow comparison, our studies largely focused on variants of such dilemmas, adapted to the animal context. However, since the original trolley scenario comes across as even more far-fetched when involving animals, most of our studies involve more realistic scenarios involving, for example, the development of vaccinations to address an epidemic, as described below.^9^ As already explained above, our main focus was on cases of intra-species sacrifice though we shall also briefly review data about inter-species sacrifice.

Across ten studies (*N* = 4,662) we found that the moral judgments of most people are best captured by MLWD. Here we will briefly summarize the key findings (for further detail, see Caviola et al., 2021).^10^

**Fig. 1:**
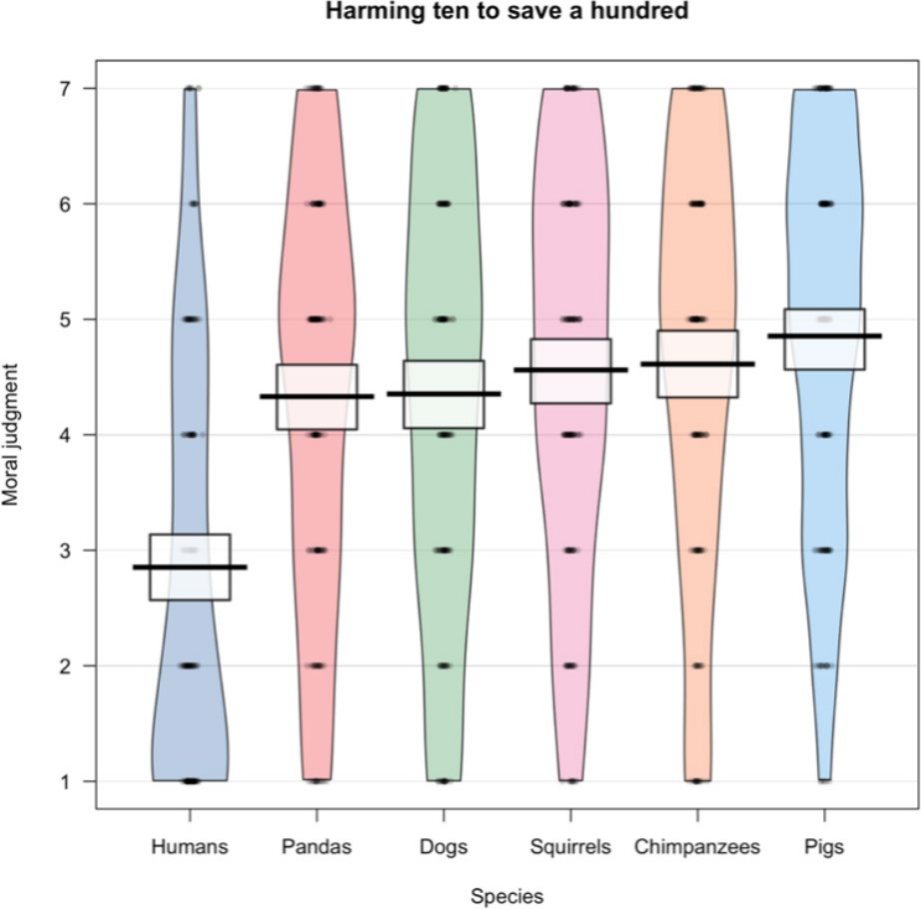
Moral judgments about harming 10 individuals of a species to saving 100 of the same species, ranging from 1 Absolutely morally wrong, to 4 Neither right nor wrong, to 7 Absolutely morally right

In one study, we presented 918 participants with a Footbridge-like sacrificial moral dilemma. Participants were asked to imagine a dilemma situation involving a sudden outbreak of a rare virus. 100 individuals were at risk of dying and the only way to save their lives was to develop a vaccine. However, in order to identify the right vaccine 10 healthy individuals of the same species, that otherwise would survive, needed to be infected, inevitably leading to their death. We manipulated the species of these two groups of individuals across conditions. In total, there were six conditions: humans, panda bears, dogs, squirrels, chimpanzees, and pigs. Thus, for example, in the pig condition, participants were asked how morally permissible they consider it to kill 10 pigs to save 100 pigs on a 7-point scale, ranging from 1 (“absolutely morally wrong”) to 4 (“neither right nor wrong”) to 7 (“absolutely morally right”). We found a strong effect between the human and the animal conditions. Participants considered it significantly more wrong to harm 10 humans to save 100 humans (*M* = 2.85, *SD* = 1.84) than to save 10 animals to save 100 animals (Fig. 1). We also found some differences between the different animal conditions. For example, participants considered it more wrong to harm 10 panda bears to save 100 panda bears (*M* = 4.33, *SD* = 1.88) than to harm 10 pigs to save 100 pigs (*M* = 4.85, *SD* = 1.69). However, these differences were rather small in comparison to the differences between the human and all animal conditions. Deontological constraints against harm were strongest for humans, followed by panda bears, dogs, squirrels, chimpanzees, and pigs.

In the same study we also asked participants how many beings would need to be saved, at a minimum, in order to make it morally right to kill 10 beings of the same species. Participants were also able to indicate that they consider it never right to make such sacrifices irrespective of the number of saved beings. We found that 65% of participants indicated that it is never right to kill 10 humans irrespective of the number of humans that could be saved. By contrast, only a minority of participants indicated that it is never right to kill 10 animals to save more animals of the same species. 34% indicated it was never right to kill 10 panda bears, 36% indicated it was never right to kill 10 dogs, 39% indicated it was never right to kill 10 squirrels, 30% indicated it was never right to kill 10 chimpanzees, and 25% indicated it was never right to kill 10 pigs. Of those participants who indicated that there was a number of saved beings that would make it morally right to kill 10 beings of the same species, the mean responses (after adjusting extreme outliers using the winsorization technique) were the following: 201 humans, 64 panda bears, 60 dogs, 59 squirrels, 53 chimpanzees, and 51 pigs.

This basic effect replicated across a range of studies and conditions: people do not shift to a purely consequentialist calculation when they consider harm to animals, even when the beneficiaries are animals of the very same species. They find such harm somewhat wrong, but still considerably less so than such harm to humans.

These findings suggest that people implicitly accept a hierarchical view on which deontological constraints get weaker as one ‘descends’ the moral hierarchy. To further explore this idea, we conducted a study to test whether people hold stronger deontological constraints for animals than for mere objects. We recruited 603 US American participants and presented them with a similar dilemma as in the previously described study, i.e., whether they consider it permissible to kill (or destroy) 10 entities in order to save (or prevent from being destroyed) 100 entities of the same type. This study had three conditions and in each condition participants were presented with two dilemmas each. In the *human* condition, the dilemmas involved human adults and children; in the *animal* condition, dogs and pigs; and in the *object* condition, paintings and chairs. For example, in one of the dilemmas in the objects condition, participants were asked how permissible they considered it to actively destroy 10 paintings if this were the only way to prevent 100 other paintings from being destroyed. We found that participants placed animals in a moral category between humans and objects. Like in the previous study, participants held stronger deontological constraints for humans (*M* = 3.28, *SD* = 2.00) than for animals (*M* = 4.70, *SD* = 1.66). But interestingly, participants seemed to even accept some deontological constraints on ‘harm’ to objects (*M* = 5.83, *SD* = 1.28), even if these were very weak (for similar findings, see Nichols & Mallon 2006). Thus, these results suggest that animals are perceived as mid-way between humans and objects in the moral hierarchy.

The studies we described thus far involved judgments about the rightness or wrongness of actions in the abstract and in hypothetical scenarios made by participants in online studies. In another study conducted off-line, we tested whether the key effect can also be observed in actual moral behaviour in a more realistic setting with first-person agential involvement of participants. For that, we recruited 208 students on the University of Oxford campus. Participants in this study were told about a planned medical research project at the university that involves developing a medicine that could help thousands of sick individuals. However, the only way to develop the medicine would be to conduct painful experiments on 50 healthy test subjects of the same species, without any long-term negative side effects. There were two conditions. In one condition, participants were told that the individuals were human infants, whereas in the other condition participants were told that the individuals were young pigs. Next participants were informed that an advocacy group had ethical concerns of this planned medical research program and was trying to prevent it. Participants were given the option of supporting the advocacy group by donating a part of their participation payment of £3 to the group. Further, they could support the campaign by voluntarily writing down the best arguments for the view that the planned research project should be prevented. They were told that we would forward these arguments to the advocacy group, which could help them improve their campaign. Thus, in contrast to the previously described studies, these participants were led to believe that their choices could have concrete impact on actual humans or animals. In the debriefing after the study, participants were informed that the medical research project was fictional. We found that the students considered it ethically more justifiable to conduct painful medical research on 50 young pigs to help thousands of young pigs than to conduct painful research on 50 human infants to help thousands of infants. Participants were more willing to invest both their personal money and their time to support a campaign against the medical research project conducted on humans than on pigs. On average, they gave 52 pennies in the human condition and only 21 pennies in the animal condition. 38% of participants were willing to write down arguments that could help improve the campaign in the human condition, whereas only 19% were willing to do so in the animal condition. These findings show that the effect we found replicates in actual behaviour in more realistic contexts.

So far, we found that people consider it more permissible to harm animals to benefit more animals than to harm humans to benefit more humans. We assumed that this was because people had weaker deontological constraints against harming animals than humans. However, an alternative explanation is that they gave greater weight to the consequences in the animal than in the human case—which would be broadly in line with the Nozickian View. In order to test which of these two moral factors is driving our effect, we conducted a study with 124 participants in which we applied what is known as ‘process dissociation’ (Conway & Gawronski, 2013; Jacoby, 1991). This technique allows us to precisely disentangle the deontological aversion to harm the few from the ‘utilitarian’ desire to help the many. We found that indeed the deontological aversion to harm animals was substantially weaker than the deontological aversion to harm humans (Cohen’s *d* = 1.15). By contrast, the ‘utilitarian’ desire to save the many did not significantly differ between the two conditions.

Although our focus was on intra-species sacrifice, in one study we also examined responses to inter-species sacrifice choices. We presented participants with the following four short questions: “Suppose you are in a situation in which you have to decide whether to kill 10 individuals to save 100 individuals. If you do nothing, the 100 individuals will die. How morally right or wrong is it to… 1) kill 10 humans to save 100 pigs, 2) kill 10 humans to save 100 humans, 3) kill 10 pigs to save 100 pigs, 4) kill 10 pigs to save 100 humans?” Using a binary response scale, we found that only 4.2% of participants considered it morally right to kill 10 humans to save 100 pigs, and 51.4% considered it morally right to kill 10 humans to save 100 humans. By contrast, 80.3% considered it morally right to kill 10 pigs to save 100 pigs, and 83.8% considered it morally right to kill 10 pigs to save 100 humans.^11^ Furthermore, we found that 83.8% of our participants indicated it would never be right to kill 10 humans irrespective of the number of saved pigs, and 46.5% indicated it would never be right to kill 10 humans irrespective of the number of saved humans. Yet only 18.3% indicated it would never be right to kill 10 pigs irrespective of the number of saved pigs, and only 14.8% indicated it would never be right to kill 10 pigs irrespective of the number of saved humans.

The results of this set of studies seem to us to provide robust support for MLWD as the descriptive view that most closely captures folk intuitions.^12^ Our findings are hard to reconcile with the competing views. Again, Utilitarianism and Cross Species Deontology can be ruled out because they assume that people attribute the same moral status to humans and animals, which is clearly not the case. Kant’s view assumes that people attribute no moral status whatsoever to animals, which can also be ruled out because people are more reluctant to harm animals more than inanimate objects. Multi-level Uniform Deontology can be ruled out because it assumes that deontological constraints against harming animals are equally strong as deontological constraints against harming humans. However, our studies showed that this is not the case: people consistently considered it more wrong to harm a few humans to help many humans than to harm a few animals to help many animals.^13^

The Nozickian View may appear to be a good first approximation of people’s intuitions. Psychologists sometimes describe judgments that see sacrificing few to save a greater number as ‘more utilitarian’ and, in that sense, people are clearly more utilitarian when it comes to animals.^14^ However, this way of interpreting such judgment is philosophically imprecise. The Nozickian View predicts that people will have no deontological constraints whatsoever against harming animals, which wasn’t the case. We found that people do have weak deontological constraints against harming animals to benefit more animals. These constraints were weaker for animals than for humans but stronger than for objects. The Nozickian View also does not explain why deontological constraints between different animal species differed—why, for example, they were stronger for dogs than for pigs. Finally, our process dissociation study found that the greater willingness to sacrifice animals was driven by a reduced inhibition to harm them, not by increased concern for consequences. Put together, these results strongly suggest that it is MLWD, not the Nozickian View, that comes closest to capturing most people’s moral intuitions.^15^

Kagan develops such a hierarchical deontological view in terms of a scale of defeasible rights of different strengths where the strength of a right is reflected by how high is amount of good (or harm prevented) that is needed to outweigh the right (Kagan, 2019, 215ff). In our studies we admittedly largely focused on the differences in participants’ ratings of wrongness. Since most philosophers reject the idea that wrongness can come in degrees, such findings need interpretation before they can be matched to ethical theories trading in categorical notions of right and wrong. We take the participants’ wrongness ratings to reflect the seriousness of the wrong and, in this way, the stringency of the corresponding deontological constraint (Hurka, 2019).^16^ However, this is not the only form of evidence supporting the MLWD hypothesis. We found the same effect in studies that also asked participants to offer binary evaluations of rightness, and as we reported above, the threshold for killing animals to save a greater number of animals was far lower compared to that for humans. And the number of participants who took an absolutist deontological stance against any such sacrifices was again dramatically lower in the animal context.

There remain important questions that require further research. For example, Killoren & Streiffer (2019) mention that most people see certain ways of treating animals as degrading and therefore unacceptable—a seemingly deontological attitude in line with MLWD. Future research could investigate whether MLWD extends to such cases and, if so, whether judgments of this sort closely align with judgments about harm of the kind we investigated—or whether the deontological constraints that people ascribe in different contexts do not add up to a coherent hierarchy. Another key issue for further research is to clarify the basis (or rather bases) for the varying deontological ‘weight’ that humans and different kinds of animals seem to enjoy. Below we review initial findings that suggest that these are not simply a function of (perceived) mental capacities or even (perceived) capacity for well-being, and that these weights reflect a variety of psychological factors, with concern for mere species membership playing a powerful role. Recent research on people’s attitudes to others’ responses to sacrificial dilemmas involving humans suggests a further possible factor: it has shown that those who engage in ‘utilitarian’ cost-benefit analysis are valued less as social partners, suggesting a possible functional explanation for the common rejection of such solutions to dilemmas (Everett et al., 2016). Future research could investigate, in line with that hypothesis, whether people endorse weaker deontological constraints in the animal case because others draw much weaker social inferences from willingness to harm animals.^17^ Relatedly, investigating whether people judge that it’s wrong to for third parties to prevent such sacrifices in the animal case will also shed light on the nature of the deontological protections that lay people ascribe to animals.^18^ Finally, since our studies also found considerable individual differences in response to sacrificial dilemmas involving animals, with at least some subjects seeming to take a more absolutist line than MLWD and others a more utilitarian line, further research is needed to attempt to more rigorously quantify the degree to which MLWD is the dominant view and to investigate the sources of this variation in views.

## Assessing the Nozickian Hybrid View

As we saw, philosophers often take the Nozickian View to be the intuitive account of the moral status of animals, and to reflect the commonsense view. And it’s easy to see why it would seem that way: most people do think that it’s permissible to sacrifice animals to benefit humans, let alone in order to save human lives, and people clearly do not ascribe to animals the strong deontological protections that they ascribe to humans. Yet when we look closely, we can see that this impression is mistaken: people do accept deontological constraints on harm to animals, it’s just that these are much weaker than in the human case. In fact, they even accept such constraints—albeit extremely weak ones—in the case of mere objects. It’s deontology all the way down.

We now turn to assess the normative implications, if any, of these findings—though repeating the caveat that this is a fairly recent area of research in moral psychology.

When Killoren and Streiffer, or Kagan, tell us that the Nozickian View should be taken seriously, one thing they might mean is that it deserves study in virtue of capturing the commonsense view, that it merits attention *because* it is the common view. This would make the project of spelling out the Nozickian View, of offering a ‘philosophical reconstruction’ of commonsense, a broadly descriptive project, even if one that is carried out from the armchair. However, if it turns out that the Nozickian View is *not* intuitive for most people, then this reason for paying it special attention is removed. Admittedly, Killoren and Streiffer also appeal to statements by figures concerned with animal welfare, the principles regulating agriculture and animal research, and so forth. To the extent that the Nozickian View does underlie such practices, it of course retains a degree of descriptive interest, and our findings do not speak to this question. We suspect, however, that a closer look will reveal either that such practices are actually better captured by Multi-level Weighted Deontology, or that these practices are themselves in part based on mistaken assumptions about the commonsense view.

On a stronger reading, the claim is rather that the being intuitive confers prima facie justification to the Nozickian View. This is in line with common practice in ethics. McMahan (2002), for example, writes that his approach assumes that.

“… unless they can be explained away as obvious products of collective self-interest, exploded metaphysics, factual errors, or some other discrediting source, our common moral intuitions should be treated as presumptively reliable, or as having some presumptive authority.” (238).

But if it turns out that the Nozickian View does not capture our common intuitions, it loses this presumptive authority. This of course doesn’t show that the Nozickian View is false, or even that it cannot be justified. But it removes one major way in which it could be justified. The question would then be whether the Nozickian View can be justified in some other way. For example, in the Kantian tradition deontological constraints are claimed to arise from autonomy or being a person or moral agent, and if (most) animals don’t possess the relevant properties, they cannot enjoy such moral protections; yet if animals still do matter morally, it seems natural to assume we should take their interests into account in a consequentialist manner.

While fully assessing such an argumentative strategy is beyond our scope here, we wish to point out the following. First, Kagan (2019) criticizes such arguments by pointing out that the candidate properties for higher moral status all come in degrees and it is therefore not clear why weaker versions of the relevant deontological protections shouldn’t be extended to animals as well. Second, such a Kantian argument arguably still relies on core intuitions about the human (or person) case—but since our findings suggest that similar intuitions extend to animals as well, this casts some doubt on the Kantian assumption that these intuitions reflect properties unique to humans (or persons). To make things worse—and this is an issue we will turn to next—some of the properties that appear to drive these intuitions are quite different from the ones Kantians postulate.

Now if common intuitive support confers ‘presumptive authority’ on a moral view, then it may rather be MLWD that enjoys such presumptive authority since it has more intuitive support than any of the competing views—though, since people respond to sacrificial dilemmas involving animals in a wide range of ways, it cannot be said that MLWD captures all, or even the vast majority, of folk moral intuitions.^19^ Given that, supporters of the Nozickian View still need to resist MLWD’s far stronger intuitive support even if they can come up with intuition-independent arguments. Moreover, as we have seen, Kagan offers powerful arguments in favour of MLWD that do not appeal to such intuitions, and this converging support suggests, at the least, that MLWD merits at least as much attention as the Nozickian View.

It might be objected that when philosophers appeal to intuitive support, they have in mind the refined intuitions of moral philosophers, not the offhand responses of bored study participants. And both Thomson and (more tentatively) Kagan report intuitions in line with the Nozickian View. Now, McMahan and Kagan explicitly refer to common moral intuitions or the commonsense moral view. Our findings directly speak to this descriptive issue. It would be interesting if the moral intuitions of moral philosophers differed from the folk on this matter. But this is an empirical question that cannot be answered by a passing remark by Judith Thomson. Moreover, Nozick, who first introduced the idea of the Nozickian View, clearly didn’t share Thomson’s intuitions; he suggests that deontological side constraints are not absolute even in the case of humans, and speculates that they may relax further in the case of animals,^20^ suggesting that his intuitions align with the majority of our participants—that is to say, with MLWD.

However, as McMahan emphasizes in the passage we quote above, if common intuitions confer authority, this authority is presumptive, and can be defeated, for example, if the intuitions merely reflect collective self-interest, or have a discredited source (see also Kahane 2011; Kahane, 2016). To address this question we turn, in the rest of the paper, to consider the implications of research we have done to investigate the sources of common intuition about the supposedly inferior moral status of animals.

## Factors driving Intuitions about Moral Anthropocentrism

People are Moral Anthropocentrists: they give clear moral priority to humans over animals (Amiot & Bastian, 2015; Caviola & Capraro, 2019; Dhont et al., 2016). In the studies we discussed so far that priority was expressed in more stringent moral constraints against harming humans. Other psychological research has also demonstrated that people are willing to harm animals to benefit humans (Awad et al., 2018; Petrinovich et al., 1993; Topolski et al., 2013). But the priority is also manifested in the context of help. For example, in one of our studies we asked 140 participants to distribute $100 to a charity that helped humans and a charity that helped animals. Both charities were presented as equally effective in reducing suffering. We found that on average participants donated $68 to the human charity and only $32 to the animal charity. In other words, people are far more willing to make sacrifices to help humans than to help animals.

So far, we were concerned with clarifying *how* people prioritise humans—we tried to identify the normative view that best captures the pattern of moral judgments that people make in this domain. Now we turn to ask *why* people exhibit this pattern of judgments—to uncover the psychological factors and processes that underlie Moral Anthropocentrism.

### Cognitive capacities and suffering

One possibility is that people attribute lower moral status to animals because they believe that animals lack certain properties that are taken to ground a higher moral status. One familiar candidate for such a property is cognitive capacity. Humans generally have far greater cognitive capacities than animals, where that includes the ability to speak and understand language, to make deliberate and autonomous judgments and to plan for the future. In philosophical parlance, possession of such higher cognitive capacities is often seen as a perquisite for being considered a person, and thus for possessing the kind of moral status that only persons are claimed to enjoy (see e.g., Kant, 1797/2017). Personhood is taken to mark a categorical distinction in moral status. But people often also seem to prioritize non-persons in line with their cognitive level, and there is evidence for such a correlation between the perceived cognitive capacity level of an individual and their perceived moral status (for a review, see Goodwin 2015; Sytsma & Machery, 2012; Piazza et al., 2014). In some of our own studies, we also examined whether the perceived difference in cognitive capacity levels between humans and animals can explain why people attribute lower moral status to animals than to humans.

We saw earlier that animals enjoy weaker deontological protections against being killed compared to humans. In a further study, we investigated whether these constraints reflect the greater cognitive capacities that humans are perceived to possess. Inspired by the so-called ‘argument from marginal cases’, we looked at people’s responses to intra-species sacrificial choices involving either animals or humans but where these were described as possessing similar cognitive capacities. This study had 103 participants, divided into two groups. We used the same vaccine dilemma that we used as in the previously described studies. One group of participants were asked whether they considered it permissible to kill 10 chimpanzees if this was the only way to save 100 chimpanzees. The chimpanzees were described as having relatively high cognitive capacities in comparison to other animals. The other group of participants were asked whether they considered it permissible to kill 10 severely cognitively impaired humans in order to save 100 severely cognitively impaired humans; these humans were explicitly described as having lower cognitive capacities than chimpanzees. We found that the participants who were given the ‘human’ dilemma considered the choice to kill some to save a greater number as more wrong (*M* = 3.64, *SD* = 1.82) than the participants who were given the ‘chimpanzee’ version of the very same dilemma (*M* = 6.72, *SD* = 1.68) even though the ratings of the cognitive capacities of the chimpanzees were higher than those of the cognitively impaired humans. In other words, most people held more stringent deontological constraints against harming humans even when these humans weren’t persons (in the philosophers’ sense) or perceived as possessing greater cognitive capacities compared to animals.^21^

Another possibility is that people attribute lower moral status to animals because they believe animals to be less capable of suffering than humans. Indeed, there is converging evidence that people attribute lower moral status to individuals the less they perceive them to be capable of suffering (Knobe & Prinz, 2008; Gray et al., 2007; Bastian et al., 2012; Bratanova et al., 2011; Loughnan et al., 2010; Kasperbauer, 2017). In one of our own studies, we tested whether people hold weaker deontological constraints against harm for animals than for humans because they believe that animals suffer less than humans. Our study had 203 participants, two conditions, and used the vaccine dilemma. In one condition, participants were asked whether they considered it permissible to kill 10 puppies to save 100 puppies. The puppies were described as having an extremely high capacity to experience pleasure and pain. In the other condition, participants were asked whether they considered it permissible to kill 10 patients in the persistent vegetative state (PVS) to save 100 other PVS patients. The patients were described as having permanently lost the capacity to experience any pleasure or pain, despite still being alive. We found that participants considered it more wrong to sacrifice PVS patients (*M* = 3.10, *SD* = 1.78) than to sacrifice the puppies (*M* = 3.80, *SD* = 1.96), despite perceiving the puppies to have higher suffering capacity than the PVS patients. Thus, perceived sentience (or more generally, potential for well-being) also doesn’t seem to underlie the stronger deontological constraints against harming humans.

These results suggest that neither cognitive capacity nor the capacity to suffer can fully explain people’s Moral Anthropocentrism. This isn’t to say that these factors have no effect on people’s moral judgments. They do: participants in our studies did tend to hold stronger deontological constraints against harm for animals, the higher they believed their cognitive capacities were and the greater their perceived capacity to suffer. But these effects were not strong enough to fully explain the difference in the strength of the deontological constraints holding in the case of humans versus in that of animals.

### Speciesism

In the previous section we saw that it is unlikely that people attribute higher moral status to humans compared to animals because they take humans to be cognitively more advanced or even to have a greater capacity to suffer. While we cannot completely rule out that there might be other morally valuable properties that humans possess but animals lack that explain Moral Anthropocentrism, we think this is rather unlikely. A simpler alternative hypothesis is that people give higher moral status to humans simply because they are members of the human species.

The tendency to give moral priority to individuals merely on the basis of species membership has been referred to as *speciesism* (Singer, 1975). Now when Singer argues that species membership alone does not justify treating humans differently from animals he is making a normative claim. But notice that the claim that Moral Anthropocentrism—both as a moral view and as a set of practices—is driven by concern with mere species membership is a testable empirical hypothesis. The studies we have reviewed above provide, we believe, robust evidence in line with this hypothesis. We found that people regard humans as deserving of greater moral protections even in cases where animals have higher cognitive capacities than humans and even when the humans in question have no capacity for suffering and, arguably, no longer have interests of *any* kind.

These are findings about the kind of factors to which moral judgements about humans vs. animals are responsive. But there is also an emerging body of evidence about the more distal causal factors that lead people to view humans are morally superior.

Some philosophers have argued that speciesism is a form of prejudice analogous to other forms of prejudice, such as racism and sexism. In prior research we found that at least psychologically there is indeed an association between speciesism and other forms of prejudice Caviola et al., 2019, 2021; for similar findings, see Dhont et al., 2016). In a study with 257 participants, we found that not only do participants differ in the extent to which they hold speciesist views, the degree to which participants endorsed such views correlated with the degree to which they exhibited racist (*r* = .32) and sexist (*r* = .41) attitudes. These associations can be explained by the fact that speciesism, racism and sexism are all expressions of a more general tendency to believe that stronger groups should dominate weaker groups and that inequality among social groups can be justified (i.e. ‘social dominance orientation’; *r* = .42). These findings indicate that the psychological basis at least of strong forms of Moral Anthropocentrism is akin to that of paradigmatic and uncontroversial forms of prejudice.

What are the origins of speciesism? Is it a hard-wired tendency that already appears in very young children or is it acquired at a later stage? In a recent study we presented 224 adults and 249 children between the age of 6 and 10 with the same moral dilemmas to compare their responses (Wilks et al., 2021). The children were recruited at various public places in the US. Both adult and children participants had to decide whether to save the life of a human or of either 1 dog, 2 dogs, 10 dogs, or 100 dogs. In another set of scenarios, we replaced the dogs with pigs but otherwise kept the scenarios identical. We found that the typical adult valued the life of a human at least 100 times more than the life of a dog, with 61% of adults saving one human over 100 dogs. When dogs were replaced by pigs, over 77% of adults saved one human over 100 pigs. By contrast, we found a strikingly different pattern in children’s responses. Children had a much weaker tendency to prioritize humans over animals: 71% of children prioritized 100 dogs over one human, and 35% even prioritized one dog over one human. Children’s tendency to prioritize humans over pigs was stronger but still substantially weaker than in adults. 53% of children prioritized 100 pigs over one human, and 18% prioritized one pig over one human. Importantly, children rated the respective cognitive and hedonic capacities of humans and animals in broadly similar ways to adults. The striking differences we found between them are therefore unlikely to be due to, say, children thinking that humans aren’t much smarter than dogs.

While these findings indicate that young children do already have a weak tendency to prioritize humans over animals, this tendency was extremely weak compared to adults. This suggests that children are far less Morally Anthropocentric than adults. And it suggests that fully blown Moral Anthropocentrism emerges relatively late in development, likely during adolescence. A recent study suggests a possible explanation for the emergence of this view. Jaquet (2019) found that when meat-eaters are confronted with ethical arguments for vegetarianism, the cognitive dissonance this produces leads them to endorse speciesist beliefs to a greater extent than meat-eaters confronted with health-based arguments. It seems likely that as older children become increasingly aware of the tension between their more egalitarian attitudes to animals and entrenched practices of meat-eating and production, they are led to rationalise the latter by letting go of the former.

### Implications for Moral Anthropocentrism

We argued earlier that if intuitive support confers presumptive authority on an ethical theory, then it is MLWD, not the Nozickian View, that has the most claim for such pro tanto justification. However, the emerging picture of the sources of common intuitions about the inferior moral status of animals is not entirely flattering. Some factors driving such intuitions reflect a concern with mere species membership—with favouring humans simply because they are humans, regardless of differences in cognition or capacity for suffering. And we further saw that there is a correlation between speciesism and paradigmatic forms of prejudice such as sexism and racism, and with the psychological traits that are typically associated with prejudice. So perhaps having this intuitive support is not such a great boon.

When Kagan (2019) develops a version of MLWD, he assumes that its decreasing levels of deontological protection (or strength of rights) would reflect decreasing levels of autonomy or some other potentially morally relevant property. Mere membership in the species *homo sapiens*, however, is unlikely to be such a property, and it is obvious enough how brute (group) self-interest would lead us to regard mere membership in *our* species as important. To the extent that the role of these factors in people’s intuitions have a debunking force, then this is a problem not just for MLWD but for Moral Anthropocentrism more generally and, perhaps, for key aspects of deontology as well.

In its strongest form, the debunking argument would go something like this:

(1) Moral Anthropocentrist intuitions reflect concern for mere species membership, and is driven by (group) self-interest, social dominance orientation and cognitive dissonance relating to meat-eating.

(2) Mere species membership, and belonging to a more powerful group are irrelevant to moral status, and judgments driven by self-interest and cognitive dissonance are unlikely to track the moral truth.

Therefore,

(3) The presumptive justification conferred by Moral Anthropocentric intuitions is defeated.^22^

But the sweeping claim in (1) is both premature and too strong. One of the lessons of the research we reviewed is that people’s moral intuitions are shaped by a multiplicity of factors and influences, and further research is needed to clarify the extent to which brute speciesism, and other biasing factors, actually drive Moral Anthropocentrism, and to what extent they might driven by factors that are potentially morally relevant. We do, however, believe that the emerging picture of the moral psychology of moral status offers support to a less radical though still important conclusion—that the degree to which most people see non-humans as morally less important reflects a range of biases, and that on a defensible Moral Anthropocentrism—one that has weeded out the influence of these biases on our intuitions—the difference in moral status between humans and other animals would be considerably smaller.

One advantage of MLWD over the Nozickian View is that it doesn’t draw a sharp normative line between humans and animals (or even persons and non-persons). Since it already construes the moral difference between humans and other animals as a matter of degree, it can more easily accommodate such qualified debunking. It’s less clear how the Nozickian View could be adjusted in light of evidence that our Moral Anthropocentric intuitions are tainted. Moreover, such a ‘correction’ of our biased intuitions can also address an important tension in the strongest current philosophical defence of MLWD. Kagan (2019) proposes such a hierarchical view of moral status on which animals matter in the *same way* as humans, yet matter *less*—and we have argued that such a view is very much in line with the dominant folk view. But Kagan *also* holds that most people are catastrophically wrong in thinking that non-human animals are *massively* less important than humans—leading to a “moral horror of unspeakable proportions”. As critics have pointed out, MLWD is perfectly compatible with such a lowly view of non-humans, and Kagan himself doesn’t really explain why (and how) this more intuitive way of interpreting MLWD is mistaken (see Fischer 2019). The qualified debunking argument we have sketched here fills this gap: allowing us to take the intuitions supporting MLWD seriously (at least provisionally) while discounting common intuitions about the distance between humans and other animals within this hierarchical view.^23^

## Conclusions

There is increasing interest in the Nozickian View and how to best develop it. This interest is often premised on the assumption that the Nozickian View just reflects common moral intuitions. The empirical evidence we reviewed here, however, suggests that another view better captures common intuitions about the permissibility of harm to humans and animals—a view we call Multi-level Weighted Deontology; it turns out that most of the folk are deontological all the way down. This of course does not mean that Multi-level Weighted Deontology is the right way to think about the moral status of humans and other animals. As we saw, there is also considerable evidence that there is a strong speciesist component to the moral intuitions that underlie Moral Anthropocentrism, the common assumption that humans morally matter more than animals. While we do not think that the currently available evidence is sufficient to entirely debunk these intuitions—and Multi-level Weighted Deontology therefore does deserve serious consideration—this evidence does suggest that we should exercise caution in simply taking these intuitions at face value. Moreover, our developmental study suggests that Moral Anthropocentrism should be malleable to social influence. A more qualified form of Moral Anthropocentrism may be within reach.^24^

## Footnotes

1 Full references here and throughout were removed to preserve anonymity.

2 Some researchers on the psychology of moral standing use ‘hybrid views’ to refer to theories that see both cognitive capacity and capacity for suffering to play a role in shaping ascriptions of moral status (see e.g. Goodwin 2015). Following Killoren & Streiffer (2019), we will be using this term here to refer only to theories that combine apply different moral principles to different classes of individuals. Since this can be done in different ways, we will refer to the Nozick-inspired approach as ‘Nozickian’—though, importantly, this *wasn’t* Nozick’s own view (see below).

3 Mulgan (2004) similarly argues that no such sharp moral distinction could be defended within a naturalistic framework.

4 Killoren & Streiffer (2019), fn. 4 raise this descriptive question about the Hybrid View.

5 To the extent that classical utilitarianism is committed to unqualified impartiality then describing the lower tier of such a two-tier morality as ‘utilitarianism’ is admittedly a bit odd. What Nozick had in mind was really the unconstrained maximising aspect of utilitarianism—the aspect that supports sacrificing some for the greater good. For a discussion of how these aspects of utilitarianism can come apart, see Kahane et al., 2018; Everett & Kahane, 2020.

6 Kagan calls such a view ‘unitarian deontology’.

7 Kagan does not endorse this view himself since he is not a deontologist.

8 For further discussion of the relation between commonsense moral judgments about cases and more explicit ethical principles and theories, see Kahane et al., (2018); Caviola et al., (2021).

9 It’s worth noting that all the studies reported here were conducted before the COVID-19 pandemic.

10 Unless otherwise specified, the participants of all the studies presented in this paper were US American citizens recruited via Amazon MechanicalTurk. To ensure reliability and validity, participants who did not pay attention or who failed to understand the instructions (as measured by attention check questions) were excluded from the analysis.

11 Since these questions were asked in the abstract, it is likely that participants accepted weaker deontological constraints against harm than in some of our other studies that involved concrete scenarios more likely to trigger affective reactions.

12 We state this so as to avoid giving the impression that MLWD is simply the folk view. While mean responses in our studies are in line with MLWD, some individuals took a more absolutist line while others’ judgments were closer to utilitarianism. But such individual differences are common in moral psychology: a large minority judges that it’s fine to throw someone off a footbridge to save five whole a sizeable minority judges that it’s *wrong* to switch to train to a side-track so that it kills one instead of five.

13 Unless we understand Multi-level Uniform Deontology to involve the same absolute prohibitions against sacrificing either humans or animals for the greater good—which would be descriptively false for most people—then it’s natural to understand it as postulating a fixed threshold of good consequences that must be met to permit such a sacrifice. A complication here is that since people also likely ascribe less value to an animal’s life than to a human’s, the consequences at stake are different in animal dilemmas than in human ones. However, if human life is more valuable, then it should be *easier*, not harder, to meet the fixed threshold in the human context. This clearly wasn’t the case.

14 Though see Caviola et al., (2021) for critical discussion of this common terminology.

15 We wrote above that our main focus was on lay people’s judgments about concrete cases, not on whether they would endorse, e.g., an explicit statement of the Hybrid View. However, in our different studies (see Caviola et al., 2021) we employed a wide variety of probes ranging from abstract questions about the value or replaceability of different kinds of beings and whether it’s ever permissible to sacrifice them to save a greater number and if so, what that number is, to imaginary dilemmas and real-life choices; all yielded similar results. In addition, participants in 6 of the studies filled out the Speciesism Scale questionnaire, whose items include fairly explicit questions about endorsement of speciesism; in 4 of these, Speciesism moderates the effect of condition (animals vs. humans) on moral judgment. In large-scale online studies it’s hard to systematically measure participants’ explicit justifications for their judgments or to allow them to consciously reflect on the consistency of their views. Interestingly, Jaquet (2019) found that when meat-eating participants were confronted with ethical arguments against meat-eating, this *increased* their endorsement of speciesist views.

16 It might be suggested that this variation in people’s judgments reflects the degree to which they see certain moral acts as morally *good* or *bad*, in the way proposed by scalar consequentialism. But it’s not clear how a scalar consequentialist view would explain why sacrificing one animal to save ten would be less bad than the parallel sacrifice in the human context (see also fn. 13).

17 Another question is whether these different factors directly shape moral judgments or whether this is mediated via the computation of some overall moral status or value; our findings don’t directly speak to this issue. We are grateful to an anonymous reviewer for raising this point.

18 Notice, moreover, that our findings only offer intuitive support to a *deontological* hierarchy—they don’t directly support the *axiological* hierarchy that Kagan (2019) also defends on which, e.g., an identical pain matters more in a human than in a non-human animal. While Kagan claims intuitive support for such difference, others deny it (see Lloyd 2021). This issue also requires further empirical research.

19 We are grateful to David Killoren and Robert Streiffer for pressing us to highlight the degree of variation in folk responses.

20 Ibid., 41.

21 In unpublished work, we found that this higher status also cannot be explained by the fact that humans are members of a *species* that *typically* has higher cognitive capacities.

22 See Jaquet (2019) for a similar debunking argument focusing only on the role of cognitive dissonance.

23 Lloyd (2021) argues that hierarchical views such as Kagan’s cannot offer a plausible account of why cognitively impaired humans with capacities comparable to some non-human animals should be placed high on the hierarchy. The findings concerning such humans we reported above suggest that intuitions about the high moral status of such humans may also need be downgraded.

24 We are grateful to two anonymous reviewers, as well as to participants in the 2019 Oxford conference on *Utilitarian Approaches in Animal Ethics*, for helpful comments, and to David Killoren and Robert Streiffer for useful criticism of our argument. Our paper draws heavily on empirical work we did in collaboration with Jim Everett, Elliot Teperman, Julian Savulescu, and Nadira Faber, as well as with Matti Wilks and Paul Bloom.
